# Characterization of structural and functional network organization after focal prefrontal lesions in humans in proof of principle study

**DOI:** 10.1007/s00429-022-02570-2

**Published:** 2022-10-07

**Authors:** Maryann P. Noonan, Maiya R. Geddes, Rogier B. Mars, Lesley K. Fellows

**Affiliations:** 1 Department of Experimental Psychology, University of Oxford, Anna Watts Building, Radcliffe Observatory Quarter, Woodstock Rd, Oxford, OX2 6HG UK; 2 Department of Neurology and Neurosurgery, Montreal Neurological Institute, McGill University, Montreal, Canada; 3 Brigham and Women’s Hospital, Harvard Medical School, 60 Fenwood Road, Boston, MA 02115 USA; 4 Centre for Functional MRI of the Brain (FMRIB), Wellcome Centre for Integrative Neuroimaging, Nuffield Department of Clinical Neurosciences, University of Oxford, John Radcliffe Hospital, Oxford, OX3 9DU UK; 5 Donders Institute for Brain, Cognition and Behaviour, Radboud University Njmegen, Nijmegen, The Netherlands

**Keywords:** Lesion, Superior frontal gyrus, Inferior frontal gyrus, Neuroimaging

## Abstract

Lesion research classically maps behavioral effects of focal damage to the directly injured brain region. However, such damage can also have distant effects that can be assessed with modern imaging methods. Furthermore, the combination and comparison of imaging methods in a lesion model may shed light on the biological basis of structural and functional networks in the healthy brain. We characterized network organization assessed with multiple MRI imaging modalities in 13 patients with chronic focal damage affecting either superior or inferior frontal gyrus (SFG, IFG) and 18 demographically matched healthy Controls. We first defined structural and functional network parameters in Controls and then investigated grey matter (GM) and white matter (WM) differences between patients and Controls. Finally, we examined the differences in functional coupling to large-scale resting state networks (RSNs). The results suggest lesions are associated with widespread within-network GM loss at distal sites, yet leave WM and RSNs relatively preserved. Lesions to either prefrontal region also had a similar relative level of impact on structural and functional networks. The findings provide initial evidence for causal contributions of specific prefrontal regions to brain networks in humans that will ultimately help to refine models of the human brain.

**Supplementary Information:**

The online version contains supplementary material available at 10.1007/s00429-022-02570-2.

## Introduction

Although it has long been known that focal brain damage can have distant effects, the neuroimaging tools needed to characterize such effects in living humans have only recently become available. Diaschisis (from Greek, meaning “shocked throughout”) refers to a loss of neuronal activity due to acute loss of afferents from a lesioned brain region and was first described by von Monakow (1914). The rapidly expanding field of network neuroscience has defined a variety of structural and functional networks and linked variation in these networks to individual differences and pathological conditions (Honey and Sporns 2008; van den Heuvel and Sporns 2013). However, the biological basis of these networks has, until recently, been inferred largely from correlational evidence from healthy people and remains a matter of debate.

In the last decade, tools developed in cognitive neuroscience have been applied fruitfully to examine the impact of lesions on structural and function networks. For example, lesion network mapping (Boes et al. 2015; Darby et al. 2018; Fox 2018) allows researchers to identify common networks from apparently spatially disparate lesions that appear to cause similar behavioral impairments by overlapping lesion volumes on a healthy connectome. While this indirect measure of network dysfunction is readily available relative to direct measures of connectivity obtained through neuroimaging of patients with brain lesions, its ability to predict behavior from functional disconnections is inferior to measures of functional and structural connectivity obtained from patients directly (Salvalaggio et al. 2020, 2021). Similarly, the predictions of network dysfunction generated from complementary in silico lesion methods (Alstott et al. 2009; Joyce et al. 2013) that create simulated lesions in healthy brain imaging scans are rarely empirically tested in lesioned patients.

Furthermore, combining human lesional approaches with several neuroimaging methodologies in the same subjects presents an important opportunity to obtain causal evidence about the contribution of a damaged brain region to underlying brain structural and functional network organization (Vaidya et al. 2019). While there are not many studies that examine the effects of focal brain lesions on structural and functional networks imaged in the same people (although see Buch et al. 2012, using MEG and DWI; Liu et al. 2016, using resting state and DWI; and indirect structural measures in Salvalaggio et al. 2020), this multidisciplinary approach can provide the route toward critical empirical validation of human in silico lesion simulation modeling techniques and ultimately can help improve and refine our ability to model the human brain (Alstott et al. 2009; Fox 2018; Reid et al. 2019; Sleimen-Malkoun et al. 2010). Accurate lesion simulation models hold promise in guiding the identification of novel, circuit-based therapeutic targets across a spectrum of neurological illness, including stroke (Fox 2018; Sleimen-Malkoun et al. 2010). With these goals in mind, we applied combined lesion and comparative neuroimaging methodology in humans with focal prefrontal damage. The current proof of principle study investigates the impact of medial and lateral prefrontal lesions on underlying structural and functional brain organization and seeks to empirically test an existing in silico model of medial and lateral prefrontal damage in humans (Alstott et al. 2009).

Specifically, Alstott and colleagues used a computational model of large-scale functional and structural connectivity, derived from diffusion spectral imaging sequences and selectively deleted nodes in different areas of the model brain (Alstott et al. 2009). The authors observed different changes in connectivity patterns depending on modality and lesion location: for example, the structural integrity of a network, defined by connectivity measures derived from WM tracts, was relatively resilient to node deletion, while node deletion had more pronounced effects on functional connectivity. Even when ‘central’ nodes were targeted, this had minimal impact on structural network integrity until over 15% of nodes were deleted. By contrast lesions that comprised only 5% of nodes had significant impact on functional connectivity that depended on lesion location. This model showed that simulated lesions along the cortical midline, including superior frontal cortex, showed profound and widely distributed impacts on network integrity. In contrast, simulated lesions involving lateral regions, including pars opercularis, resulted in reduced connectivity more locally (Alstott et al. 2009).

In the present study, we acquired structural and resting state functional MRI data in the same sample of patients with chronic frontal lobe lesions involving either superior frontal gyrus (SFG) or right inferior frontal gyrus (rIFG). These two lesion sites overlap with adjacent simulated lesion sites in Alstott et al. (2009), described there as superior frontal cortex and pars opercularis, respectively. We focus on the structural and dynamic impacts of prefrontal lesions rather than brain-behavior relationships. The behavioral impact of these lesions on cognitive control and behavioral inhibition has been characterized elsewhere (Geddes et al. 2014; Modirrousta and Fellows 2008; Tsuchida and Fellows 2009; Yeung et al. 2021). Here, we assessed lesion effects on grey matter (GM), WM and resting state functional connectivity, comparing lesion groups to healthy Controls. We applied the standard FSL analysis platforms of voxel-based morphology (VBM), tract-based spatial statistics (TBSS) and dual regression analysis to structural scans, diffusion weighted MRI scans and resting state scans, respectively. Indeed, the current understanding of the impact of frontal lobe lesions on inter-network connectivity is particularly unclear, with evidence both for and against lesions effects on functional connectivity among undamaged network nodes (Eldaief et al. 2017; Nomura et al. 2010). Here, we ask what, if any, is the impact of a SFG or rIFG lesion on brain networks identified through structural or resting state functional neuroimaging. Based on simulated lesion results (Alstott et al. 2009), we predicted that lesions would affect functional connectivity to a greater extent than structural white matter connectivity. Alstott did not simulate the impact of lesions on distal GM, but we again speculated that lesions would affect functional connectivity more than structural GM anatomy.

The primary objectives of the current study are threefold. We sought to (1) empirically test an existing computational model in humans by applying an inferentially powerful direct method, (2) characterize resilience and vulnerability across structural and functional brain networks following chronic prefrontal damage, and, (3) identify whether any observed impacts differ by lesion location. While this study will not examine the link between connectivity changes and behavior, it will contribute direct network measurement using some of most well-established neuroimaging analysis pipelines, which will ultimately lead to improved models of the human brain.

## Methods

### Participants

Fourteen people with focal lesions involving the frontal lobes were recruited from the Cognitive Neuroscience Research Registry at McGill University. Individuals with prefrontal damage were eligible if they had a focal damage affecting one of the frontal lobe regions of interest: rIFG or SFG. One participant could not complete the MRI due to joint pain, leaving 13 participants as the final sample (8 women; mean age (standard deviation (SD)) = 58 (12.9) years). Age- and education-matched healthy Control participants (*n* = 18, 11 women; mean age (SD) = 51.9 (15.3) y) were recruited through local advertisement in Montreal. They were free from neurological or psychiatric disease and not taking psychoactive medication. Demographic information is reported in Table 1. Controls completed screening tests for mild cognitive impairment and depression. All scored 26 or greater on the Montreal Cognitive Assessment (MoCA) (Nasreddine et al. 2005), and less than 12 on the Beck Depression Inventory. Patients completed additional neuropsychological screening to test memory, language, attention and executive functions at the time of enrolment in the Registry (Table 2). Lesion groups were compared using independent samples *t* tests. Lesions were due to ischemic stroke (*n* = 7), low-grade tumor resection (*n* = 5), fast-growing glioma (*n* = 1). The median time since the lesion occurred was 5 years (range 1–10 years). All subjects provided written informed consent in accordance with the Declaration of Helsinki and were compensated for their time with a nominal fee. The study was approved by the MNI’s research ethics board.

**Table 1:** Demographic information

Group	Age (years)	Education (years)
SFG	59.7 (11.7)	15.1 (2.7)
rIFG	55.8 (15.2)	13.9 (4.4)
Controls	52.9 (15.3)	15.8 (3.4)

Data are mean (SD)

**Table 2:** Neuropsychological screening

Group	BDI	IQ estimate (WASI)	Animal fluency	F-A-S fluency	Backwards digit span
SFG	9 (11)	113 (7)**	17 (5)	36 (19)	5.9 (2.1)
rIFG	13 (6)	109 (13)*	16 (4)	34 (19)	6.6 (2.3)*

Data are mean (SD)

*1 participant missing

**2 participants missing

**Fig. 1:**
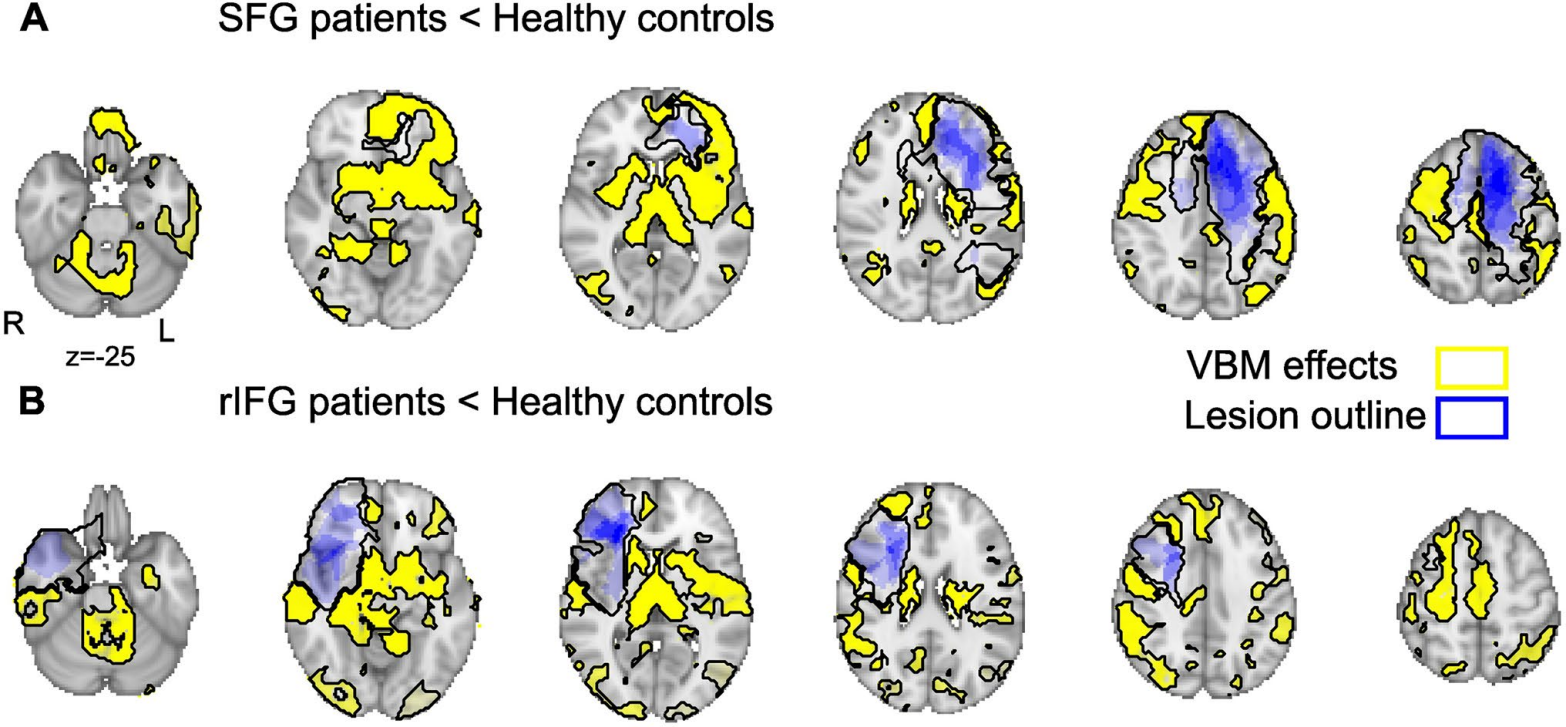
Lesion overlap (blue; intensity reflects number of patients) and VBM lesion effects (yellow) for Controls > SFG (**A**) and Cont > rIFG (**B**). Brain slices increase by internals of 14 mms from the most ventral slice of *z* = − 25

Lesions were traced from the available CT or MRI onto the standard Montreal Neurological Institute (MNI) brain using MRIcro software. In two cases, lesions were labelled manually on a T1-weighted MPRAGE image prior to registration to the MNI brain. All lesions were overlaid to define the cluster of maximum overlap (lesion overlap image files are available in supplementary material). In the 7 patients with SFG damage, the center of gravity of this cluster fell within dorsomedial WM (− 15, 11, 50). The overlap in all 6 rIFG patients was also in WM (28, 31, 11). No patient had an overlap of lesions that included both brain areas. Figure 1 shows the overlap image of lesion tracings for each group. We calculated the maximal number of patients with voxels damaged within a lesioned area. Despite variability in lesion location and extent, voxels in a cluster sized 3704 mm^3^ were damaged in all SFG patients. For the rIFG group, all lesions overlapped in a 560 mm^3^ WM cluster. The two lesion groups did not differ in lesion volume (*t*_11_ = 1.67, *p* = 0.124). All GLMs described below included sex, age, handedness and number of years of education as confound regressors. The variance associated with these latter variables is therefore effectively regressed out of the group-wise effects.

### Image acquisition

All images were acquired on a 1.5 T Siemens MR scanner at the McConnell Brain Imaging Centre at the Montreal Neurological Institute, McGill University. Participants laid supine in the scanner, and cushions were used to reduce head motion. BOLD fMRI data were acquired using echo planar imaging (EPI) (36 × 4 mm thick axial slices with a base resolution of 64 mm, field of view 256 × 256 x 144mm^3^, giving a voxel size of 4 × 4 x 4 mm, repetition time = 2.8 s, 153 volumes, echo time = 50 ms, and flip angle = 90°). The EPI scanning sequence lasted 7 min 20 secs, and participants were instructed to keep their eyes closed, not to think of anything in particular. A T1-weighted anatomical image was acquired for each participant (repetition time = 2800 ms, echo time = 4.12 ms, and flip angle = 15°, giving a voxel size of 1 × 1 × 1 mm). Diffusion MRI (DMRI) data were also acquired from 17 of the same participants described above with the same scanner. A technical issue meant it was not possible to collect the DMRI in the 18th Control participant. DMRI data were acquired using echo planar imaging (75 slices, 2 mm thick axial slices; field of view, 256 × 256 × 150 mm; giving a voxel size of 2 × 2 × 2 mm). Diffusion weighting was isotropically distributed along 99 directions using a B value of 1000 mm^2^. Ten volumes with no diffusion weighting were acquired throughout the acquisition. The total scan time for the DMRI protocol was 20.21 min. Note, the data that support the findings of this study are available from the corresponding author upon reasonable request.

### Preprocessing

Data were analyzed using tools from the FMRIB Software Library (www.fmrib.ox.ac.uk/fsl). All structural and EPI images were converted to nifti and skull stripped with BET. In instances when the automatic brain extraction tool made localized errors, more likely in an older and lesioned sample, we corrected the brain mask by hand; reinstating brain voxels into the brain mask image or removing non-brain voxels from the brain mask.

### Structural reorganization after frontal lesions

We examined GM and WM differences between lesion groups and Controls using voxel-based morphometry (VBM) and tract-based spatial statistics (TBSS).

#### Voxel-based morphometry

We used voxel-based morphometry (Douaud et al. 2007http://fsl.fmrib.ox.ac.uk/fsl/fslviki/FSLVBM) to identify areas of GM where volume differed by group. The skull stripped T1-weighted structural images were individually segmented into GM, WM and cerebrospinal fluid before being affine-registered to the GM ICBM-152 template using FLIRT (Jenkinson and Smith 2001) followed by nonlinear registration using FMRIB’s Nonlinear Image Registration Tool (FNIRT) (Andersson et al. 2010). A study-specific template was created using six participants from each group so as not to bias the structural template toward the groups with the larger number of participants (i.e., Controls). Each group was represented equally in the template and contributed the same number of T1 scans as the smallest sized group. Scans were chosen randomly from the larger groups. All native GM images from the whole sample were then nonlinearly re-registered and concatenated into a 4D image which included Controls, SFG and rIFG patients. The registered partial volume images were then modulated (to correct for local expansion or contraction) by dividing by the Jacobian of the warp field. The modulated segmented images were then smoothed with an isotropic Gaussian kernel with a sigma of 3 mm. Segmentation and registration was confirmed by visual inspection.

The resulting 4D image was then used within two independent analyses based on a single GLM. The GLM was identical for each analysis with each patient group and Controls indexed as three separate regressors. The model was implemented using permutation-based nonparametric testing with Randomize (*n* = 5000), corrected for multiple comparisons (*p* < 0.05) over a GM mask with threshold free cluster enhancement (tfce) methods (Smith and Nichols 2009). Each analysis differed only by the group contrasts and the GM inclusion mask. Specifically, Controls and SFG patients were compared in one analysis excluding any SFG lesion damaged voxel. Controls and rIFG patients were compared in another analysis excluding any rIFG lesion damaged voxel. Damaged voxels were removed from the analyses by using the respective lesion overlap masks as an exclusion mask. We examined positive and negative group contrasts in each analysis, primarily each lesion group to the larger sample of healthy Controls. For completeness, we also compared SFG and rIFG patients directly excluding any voxel within either groups’ lesions.

### Tract-based spatial statistics

Next, we assessed the impact of lesions to the structural integrity of WM by measuring differences in fractional anisotropy. We assessed group differences in WM integrity with the FSL TBSS processing pipeline (Smith et al. 2006http://fsl.fmrib.ox.ac.uk/fsl/fslwiki/TBSS). Specifically, the pre-processed data were subjected to DTIFIT, an analysis step which fits a diffusion tensor model at each voxel in order to generate a 3D fractional anisotropy image for each participant. This image was registered to the FMRIB58FA standard brain before a study specific skeletonized FA template was generated and thresholded at 0.2. All participants’ skeletonized FA images were concatenated into a 4D image which included Controls, SFG and rIFG patients.

We focused exclusively on the WM tracts identified in healthy Controls as emanating from the lesion sites and terminating in grey matter that overlapped with the VBM lesion effects (see Supplementary Methods: Probabilistic tractography). The probtrack group tractograms seeded bilaterally at the lesion coordinates in healthy Controls were used as small volume of interest masks to constrain the voxel-wise group statistics that used nonparametric permutation testing (Nichols and Holmes 2002) with Randomize (Winkler et al. 2014). The GLM included factors of Group, with each patient group and Controls indexed as three separate regressors, as well as the confound regressors. In two separate analyses, we examined the Patient (SFG or rIFG) < Control contrast. All reported statistics were found to survive cluster correction for multiple comparisons (*p* < 0.05) with tfce methods (Smith and Nichols 2009). Again, any lesioned voxel was excluded from the statistical tests by using the lesion overlap mask as an exclusion mask. For visualization and identification of WM tracts identified by the TBSS analysis, we ran probabilistic tractography seeded from 216 mm^3^ masks placed at the center of gravity of each cluster. Probabilistic tractography parameters and details related to the construction and post-processing of tractograms were identical to those used in the probabilistic tractography analyses in healthy Controls and are described in Supplementary Methods: Probabilistic tractography.

### Structural connectivity of lesion sites

In a series of complementary connectivity analyses in healthy Control data, we first defined the lesion site by its structural connectivity. Using probabilistic tractography seeded from the WM lesion overlap coordinates in healthy Control participants, we estimated connectivity to cortical and subcortical GM anatomical targets. Subsequently, we utilized this data to confirm that the network identified by the VBM analysis above in patients is what we might expect from transneuronal degeneration from the lesion site. We therefore concurrently examined connectivity of the two lesion sites in the healthy Control group, as well as a non-lesioned Control site in the patients. We chose to compare against a control site to remove the possibility of biasing the results if one lesion location had a broader connectivity pattern than another. Specifically, we conducted two additional connectivity analyses in the healthy Control sample: probabilistic tractography and seeded resting state. These analyses are described in Supplementary Methods: Probabilistic tractography and Seeded resting state.

### Functional reorganization of resting state networks after frontal lesions

#### Dual regression

Finally, we examined the impact of the two lesions on functional resting state networks. Each participant’s individual functional EPI data were first preprocessed using Multivariate Exploratory Linear Optimized Decomposition into Independent Components (MELODIC). Components that were clearly caused by head motion or spikes were removed.

Resting state functional connectivity was assessed using the dual regression technique (Filippini et al. 2009http://fsl.fmrib.ox.ac.uk/fsl/fslwki/DualRegression). This three-step method allows for voxel-wise comparisons of resting state network functional connectivity. We examine the differential contribution of voxels in the brain to large-scale RSNs between patients and Controls and as such our voxel-wise methods are equivalent across GM, WM and functional connectivity. First, all participants’ denoised resting-state functional MRI (rsfMRI) data were collectively motion corrected, spatially smoothed (using a Gaussian kernel of full-width at halfmaximum (FWHM) of 6 mm) and high-pass temporally filtered to 150 s (0.007 Hz). Individual fMRI volumes were registered to the individual’s structural scan and standard space images using FNIRT. Pre-processed functional data containing 154 time points for each Control participant were temporally concatenated across participants to create a single group 4D FMRI data set. Note, no lesioned data were included in this initial ICA decomposition. This concatenated group data set is then decomposed using independent component analysis (ICA). ICA is a data-driven approach used to identify large-scale patterns of functional connectivity in the healthy population of participants. In this analysis, the data set was decomposed into 20 components, in which the model order was estimated using the Laplace approximation to the Bayesian evidence for a probabilistic principal component model.

The second step uses the dual-regression approach to identify, within each participant’s fMRI data set, subject-specific temporal dynamics and associated resting state network (RSN) spatial maps. This step was run on all lesion and Control participants and involves (1) using the full set of group-ICA spatial maps in a linear model fit (spatial regression) against the separate fMRI data sets, resulting in matrices describing temporal dynamics for each component and participant, and (2) using these time-course matrices in a linear model fit (temporal regression) against the associated fMRI data set to estimate subject-specific spatial maps. For each patient, the individual lesion site was masked so that BOLD signal variance in these voxels did not contribute to this second step.

We focused on RSNs, defined by the Yeo atlas (Yeo et al. 2011), that were structurally most affected by the lesions. We calculated the degree of spatial overlap between individual patients’ lesions and the RSN normalized for the total spatial extent of each RSN. From the seven Yeo RSNs, we could identify six networks through spatial overlap extent within our 20 ICA components (the Limbic network could not be identified) and we determined the top two spatially coincident RSNs in each lesion group and compared the degree of lesion overlap of each individual across the two patient groups with independent samples t-tests. On these six RSNs, we performed the third and final step of the dual regression pipeline. The RSN component maps were concatenated across all participants into single 4D files (1 per original ICA map, with the fourth dimension being subject identification) before being subjected to nonparametric permutation testing (randomize *n* = 10,000). Again cluster-based thresholding (clusterm *c* = 2.3, *p* < 0.05) (Nichols and Holmes 2002) was calculated over a small volume correction GM mask. This mask excluded lesioned voxels, indexed by the lesion overlap mask, and was restricted to voxels that fulfilled either of the following criteria [1] the spatial extent of the RSN of interest or [2] the spatial overlap between VBM lesion effects and sRS network in healthy Controls (see Supplementary Methods: Seeded resting state). As such, even though the GLM was the same, with each patient group and Controls indexed as three separate regressors, Controls were compared to patient groups in two separate randomize analyses with different GM inclusion masks. For each patient group separately, we tested two contrasts; Controls > Lesion group and Lesion group > Controls. For illustration, all effects were then up-sampled to 2 mm^3^ resolution.

## Results

### Participant characteristics

Demographic information and neuropsychological screening test results are provided in Tables 1, 2, respectively. Controls were similar to both patient groups in age (*p* values =  > 0.236) and education (*p* values =  > 0.291). Further, the patient groups did not differ on the Beck Depression Inventory (*p* = 0.432), estimated IQ (*p* = 0.500), Animal Fluency (*p* = 0.666), F-A-S Fluency (*p* = 0.851) or backwards digit span (*p* = 0.576).

### Defining the lesion site

As the maximal site of lesion overlap for both groups was in WM (Fig. 1), we used connectivity analyses to determine the cortical structures most likely affected by these lesions. Using probabilistic tractography seeded from the WM lesion overlap coordinates in healthy Control participants, we estimated connectivity to cortical and subcortical GM anatomical targets. This analysis is the first step in a series that defines the network and network parameters of the SFG and rIFG in healthy Controls. Analysis details are provided in Supplementary Methods: Network definition and parameter measures in healthy Controls. Results, shown in Supplementary figure S4A, suggest that the SFG WM seed is most structurally connected with the SFG, but also to a lesser extent with the MFG and cingulate regions. The rIFG WM seed is most connected with the IFG (pars triangularis) but also closely coupled with frontal polar cortex.

### Extended differences in structural morphometry beyond the lesion sites

We performed a voxel-based morphometry (VBM) analysis to identify the extended GM network altered by SFG or rIFG lesions relative to Controls. As illustrated in Fig. 1, GM differences extended well beyond the lesion site. Our results show that SFG patients have reduced GM relative to Controls in superior frontal gyrus, caudate, thalamus and frontopolar cortex and insula (full VBM contrast maps are available in supplementary material).

RIFG patients showed reduced GM relative to Controls in several brain regions that include temporal cortex, temporoparietal cortex, middle, and inferior, frontal gyrus, caudate, thalamus, brainstem, cerebellum and orbitofrontal cortex (see VBM contrast maps). The total number of voxels significantly different relative to Controls were similar in the two groups (SFG = 48,211 and rIFG = 49,479). No clusters in which GM was larger in patients compared to Controls survived correction for multiple comparisons. Furthermore, we found no VBM differences between SFG and rIFG patients, although we note that we are only able to examine 26% of the brain in this analysis because of voxel damage in any individual patient.

### Connectivity of the SFG and IFG in healthy controls

The aim of the next analyses was to confirm that the network identified by the VBM analysis in patients is what we might expect from transneuronal degeneration from the lesion site. We performed two connectivity analyses in healthy Controls using DMRI and resting state data to define the normal connectivity of each lesion site.

First, we seeded probabilistic tractography in WM within the two lesion sites, and a control site in lateral occipital fusiform gyrus (LOFG; see Supplementary Methods: Probabilistic tractography). Tractograms and WM projections are illustrated and described in Supplementary results are shown in Figure S1A–C. We quantified the degree of overlap between the DMRI and VBM lesion-derived networks by using the VBM effects as classification masks and taking the median connectivity value between each WM ROI seed and all significant VBM voxels in each participant. As expected, tracts seeded from the SFG WM seed reached the SFG VBM lesion affected regions more compared to a control LOFG WM seed (Supplementary Figure S1D, SFG v LOFG *t*_16_ = 6.39, *p* < 0.001). Similarly, as predicted, tracts seeded from the rIFG were more likely to connect with IFG VMB lesion effects than the control LOFG WM seed (Supplementary Figure S1E, IFG v LOFG *t*_16_ = 10.75, *p* < 0.001).

**Fig. 2:**
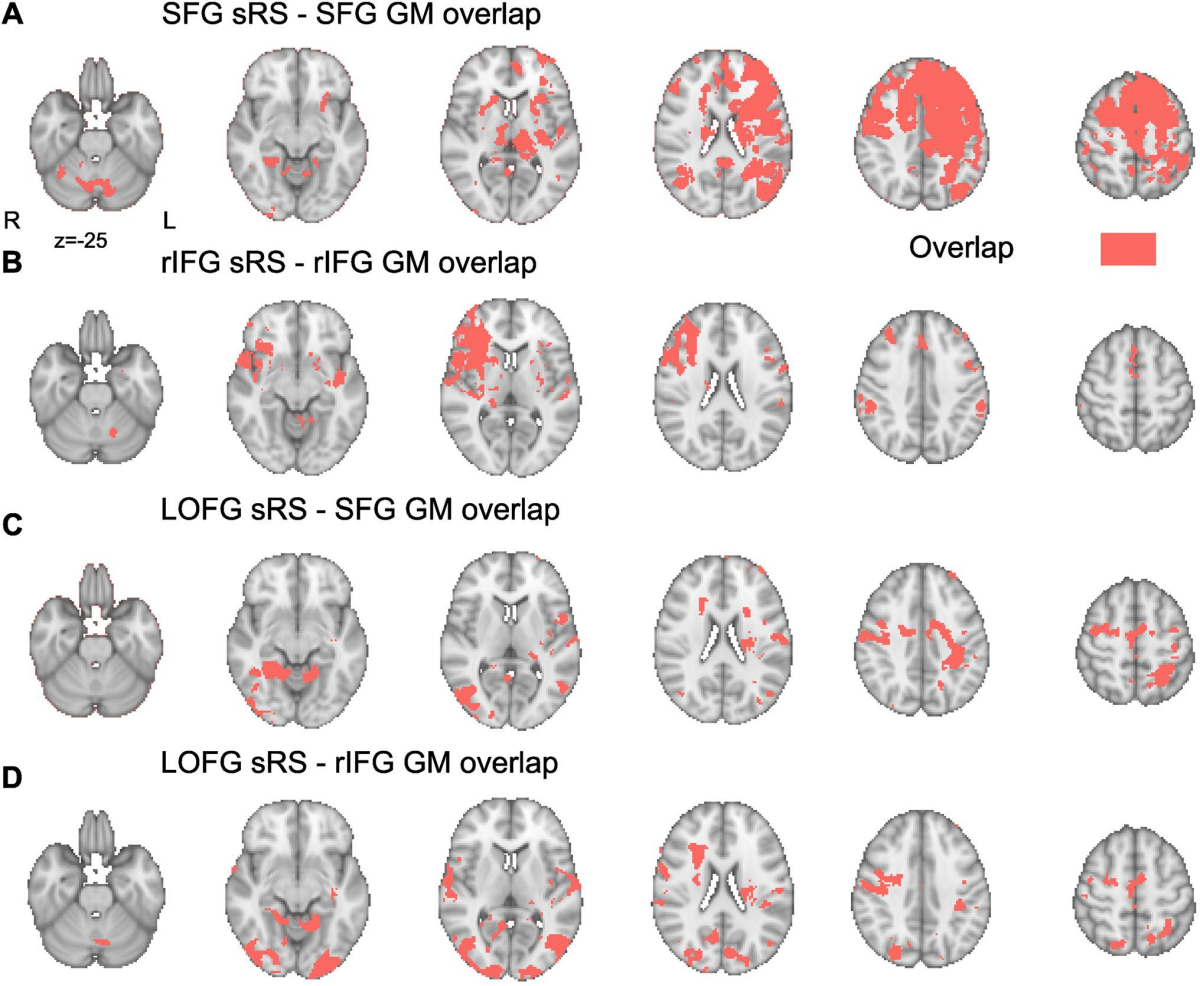
Seeded resting state analysis in healthy Controls seeded from center of gravity of the overlap shown of **A** seven SFG patients and **B** six rIFG patients. The SFG seed was moved laterally into the closest GM shown overlapped (red) with the SFG VBM lesions effects (yellow in Fig. 1). Seeded resting state from SFG lesion location shown in green in supplementary Figure S2. rIFG seed was moved laterally and ventrally into the closest GM voxels shown overlapped with the rIFG VBM lesions effects (yellow in Fig. 1). **C**, **D** show seeded resting state analysis in Controls seeded from the lateral occipital fusiform gyrus (green in supplementary Figure S2) shown overlapped with the **C** SFG VBM lesions effects and **D** rIFG VBM lesion effects. Brain slices increase in intervals of 14 mm from the most ventral slice of *z* = − 25

Second, again in healthy Controls, we seeded separate resting state analyses in GM coordinates of the SFG and rIFG lesion sites (see Supplementary Methods: *Seeded resting state)*, and the LOFG as a control site. We identified regions that were significantly coupled to the SFG, rIFG and LOFG seeds and compared the spatial topography of these seeded resting state (sRS) networks (shown in Figure S2) with the topography of the VBM lesion effects (Fig. 1). The results show that the SFG VBM lesion effects overlap with the functionally defined sRS network defined in the healthy brain (Fig. 2A; see supplementary results file sRSSFGcoord_Control_thresh_zstat.nii.gz for thresholded resting state contrast map). In addition to the dorsomedial cortex directly affected by the lesion, the functional connectivity network overlap extends beyond the lesion into other regions including contralesional SFG, medial frontal gyri, pre- and post-central sulcus, insula, as well as bilateral rostral frontal polar cortex and lateral occipital cortex. The analysis also reveals overlap in the thalamus, putamen and hippocampus (see supplementary results file LesionVBMSFG_x_sRSSFG.nii.gz for full effects. Note to reproduce the overlap effects the VBM contrast maps, e.g., VBM_Control > SFG_Corrp_tstat.nii.gz must be overlaid with the Lesion overlap images SFGLesionOverlap.nii.gz).

rIFG VBM lesion effects also overlap with the rIFG sRS defined network (Fig. 2B; see sRSrIFGcoord_Control_thresh_zstat.nii.gz). As well as functional overlap within damaged cortex, the functional network extends and overlaps beyond the lesion site to contralesional IFG, bilateral paracingulate cortex and medial SFG/SMA, Planum Polare, supramarginal gyrus and superior temporal gyrus, as well as subcortically in the putamen (see results file LesionVBMrIFG_x_sRSrIFG.nii.gz for full effects). The control analysis, seeded in the LOFG (sRSLOFGcoord_Control_thresh_zstat.nii.gz), overlapped to a lesser degree with either VBM lesion effects (Figure S2C, D, see results file LesionVBMSFG_x_sRSLOFG.nii.gz and LesionVBMrIFG_x_sRSLOFG.nii.gz for full effects).

We quantified the degree of overlap between sRS and VBM lesion-derived networks, using the Harvard Oxford parcellation atlas (See Appendix Harvard Oxford Atlas) to identify the proportion of ROIs (excluding those affected by the lesion) in which significant sRS effects for each network overlapped with significant VBM effects (Supplementary Table S1). As expected, as a proportion of size of the sRS network, both sRS SFG and sRS IFG network overlapped with their respective VBM networks to a greater degree than the sRS LOFG control network. In the SFG case, over 100% more VBM ROIs overlapped with a SFG sRS network than LOFG sRS network. In the rIFG case, 29% more VBM ROIs overlapped with a rIFG sRS network than LOFG sRS network. These independent analyses in healthy Controls provide evidence that lesions to these two regions likely result in distributed changes at distal GM regions identified in the VBM analyses. The WM tracts and sRS networks defined in healthy Controls were used to statistically constrain later analyses in the lesion groups.

### Limited differences in structural connectivity of WM tracts after frontal lobe lesions

**Fig. 3:**
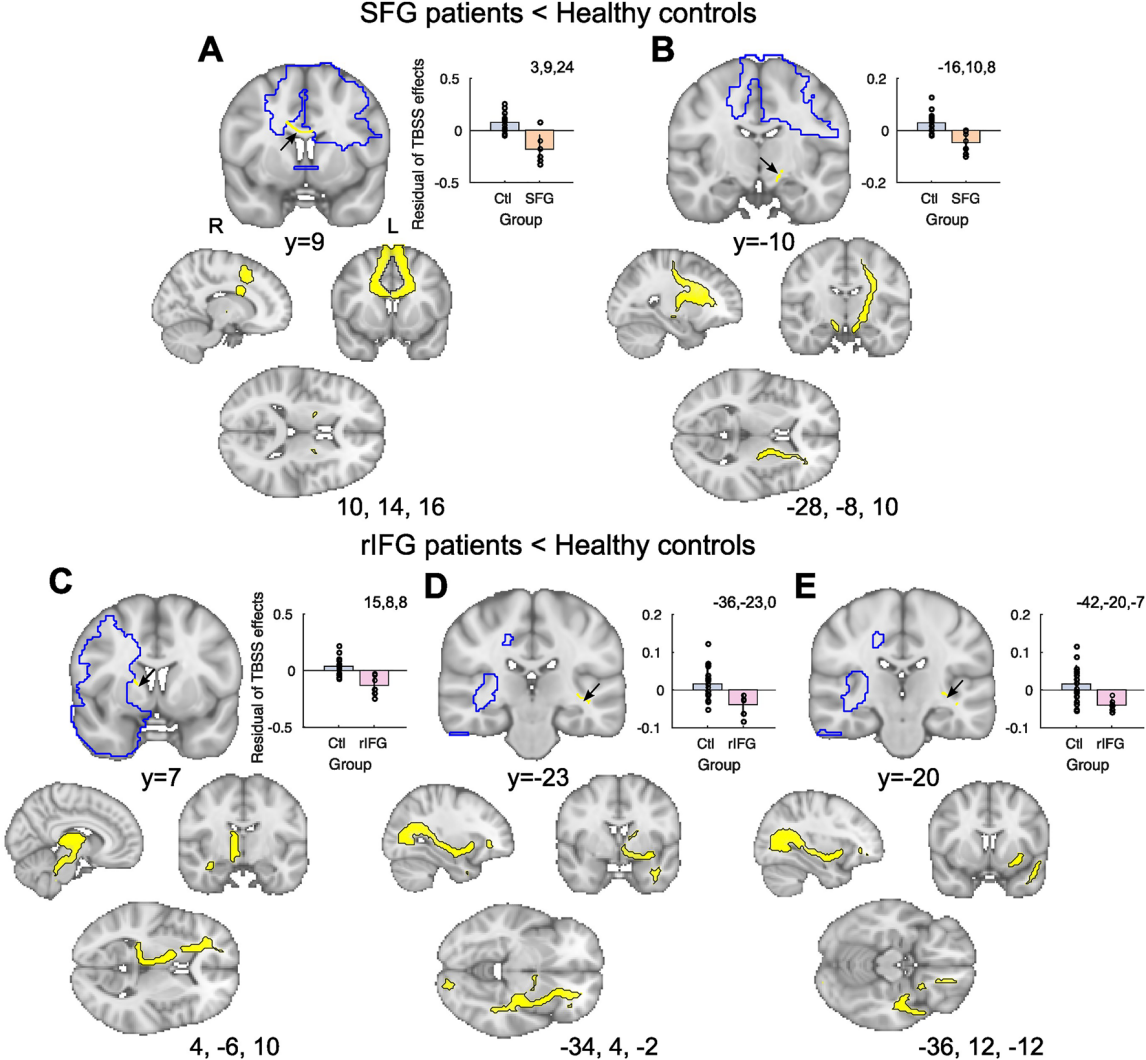
Effects of superior frontal gyrus (SFG) and right inferior frontal gyrus (rIFG) lesions on fractional anisotropy (FA). TBSS results show reduced FA in the corpus callosum and contralesional cerebral peduncle after SFG lesions (arrows in **A**, **B**), and reduced FA in bilateral internal capsule and contralesional ILF/IFOF after rIFG lesions (only illustrating right hemisphere effects; arrows in **C**, **D**, **E**). For illustration, we show residual TBSS WM effects extracted from clusters identified in the group level lesion contrasts in the bar plot insets. Lower panels: respective tracts were visualized with probabilistic tractography seeded from the TBSS effects (depicted in yellow)

Next, we analyzed the impact of lesions to the structural integrity of WM by measuring differences in fractional anisotropy. We performed a TBSS analysis to identify the WM network altered by SFG or rIFG lesions relative to Controls. Note, the analyses were constrained to the tracts identified using probabilistic tractography in healthy Controls as emanating from the lesion site coordinates. Unlike GM lesion effects, WM effects were relatively limited and often localized close to the lesion site (Fig. 3). Our results show that SFG patients show reduced WM relative to Controls in the corpus callosum (629 voxels, MNI: 5, 15, 19) and contra-lesional cerebral peduncle (74 voxels, MNI: − 13, − 9, − 8).

By contrast, rIFG patients show reduced WM FA relative to Controls in bilateral internal capsule (which included the retrolenticular portion and Extreme Capsule (EmC) in the contralesional hemisphere 350 voxels, MNI: 19,10,9 and 49 voxels, MNI: − 32, − 23, 0), as well as contralesional ILF/IFOF (Inferior longitudinal fasciculus/inferior fronto-occipital fasciculus; 41 voxel, MNI: − 40, − 22, − 8). Tracts were visualized with probabilistic tractography seeded from the TBSS effects (lower panels) and identity confirmed with the Xtract WM atlas. No WM clusters survived correction for multiple comparisons in a contrast examining greater FA in Lesioned Groups compared to Controls.

### Limited differences in resting state networks after frontal lobe lesions

**Fig. 4:**
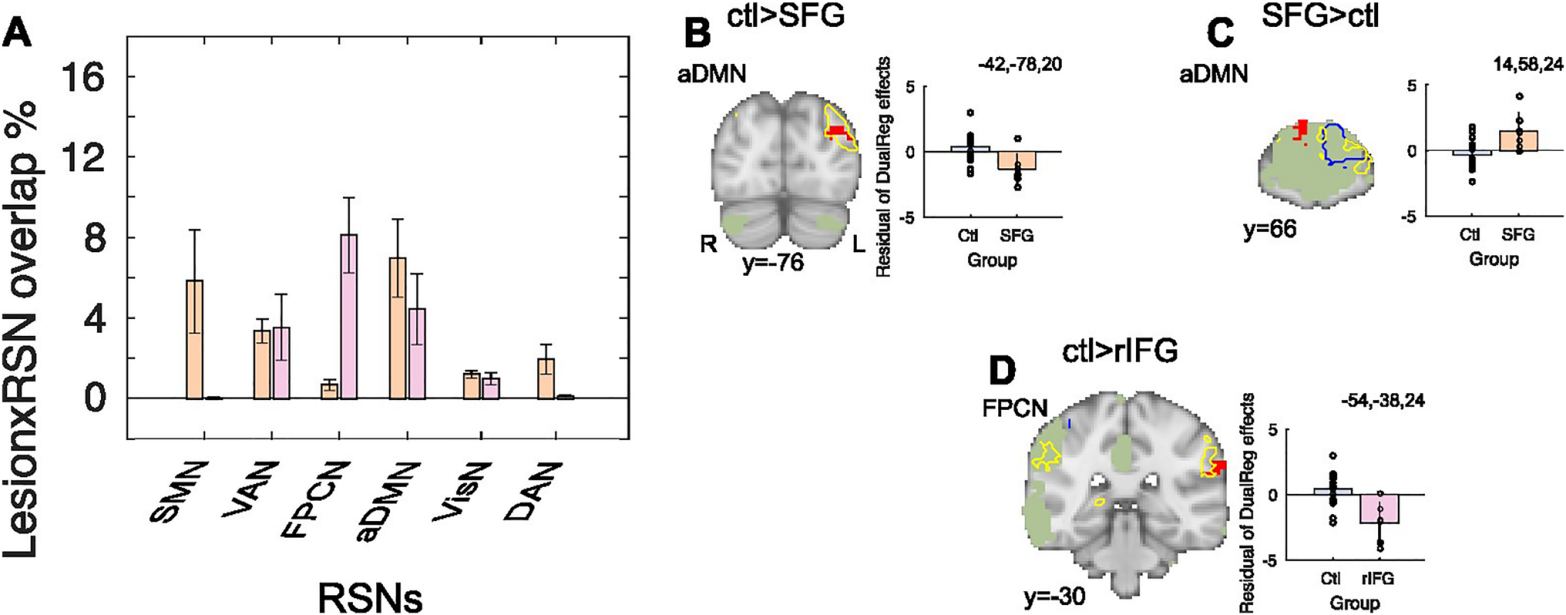
Lesion effects on resting state networks (RSNs) functional connectivity. **A** Proportion of voxels that overlap between of each of the six Yeo RSNs and each patients’ lesion mask for the SFG (peach) and rIFG (pink), respectively. The SFG lesions overlap most with the Sensorimotor Network (SMN) and anterior Default Mode Network (aDMN). The right inferior frontal gyrus (rIFG) overlap most with the Frontoparietal control network (FPCN), and the aDMN. **B**–**D** SFG patients show reduced functional connectivity between left lateral occipital cortex connectivity with aDMN (**B**) and increased functional connectivity between right rostrolateral frontal polar cortex connectivity and aDMN (**C**). rIFG patients show decreased functional connectivity between FPCN and left supramarginal gyrus cortex connectivity (**D**). Residual dual regression effects extracted from clusters identified in the group level lesion contrasts are illustrated in bar plot insets. Total lesion outline represented in blue, outline of overlap between VBM GM effects and seeded RSN in yellow, ICA network in green, dual regression effects in red

The final key analysis investigated functional differences within RSNs between Lesion Groups and Controls using dual regression methods. We hypothesized that key resting state networks putatively affected in the SFG and rIFG groups should be those in which lesions were most coincident with the spatial topography of RSNs (defined from the Yeo atlas). Overlap of individual lesion extents with each of the six Yeo networks is shown in (Fig. 4A).

We primarily focused on two networks that overlap most with each lesion group. For the SFG group, these were the anterior component of the default mode network (aDMN) and somatomotor network (SMN). For the rIFG group, the RSNs were the frontoparietal control network (FPCN) and aDMN. We hypothesized that these RSNs would show altered functional connectivity in the respective lesion groups relative to Controls. As expected, given our methods of selection, the SMN most overlapped with the lesions of those in the SFG group (*t*_11_ = 3.31, *p* = 0.007) and the FPCN (*t*_11_ = − 4.29, *p* = 0.001) overlapped most with the lesions in the rIFG group. However, the relative overlap between the lesion groups and the aDMN did not differ (aDMN: *t*_11_ = 0.96, *p* = 0.356).

Given the wide extent of the GM differences observed in both lesion groups, we found surprising conservation of functional networks. However, there were some local differences in connectivity within some networks. First, comparisons between SFG patients and Controls revealed that the ipsilesional left lateral occipital cortex showed reduced functional coupling with the aDMN in SFG patients (Fig. 4B, *p* = 0.006). In the reverse contrast, there was more functional coupling between the aDMN and the contralesional right rostrolateral frontopolar cortex in SFG patients (Fig. 4C, *p* = 0.013).

For completeness, we also examined the impact of SFG lesion on functional connectivity in the other RSNs. We identified two other contrasts that showed significant differences in functional connectivity between SFG patients and Controls; with more functional connectivity between the ipsilesional left insula cortex and the VAN (*p* = 0.026), and more contralesional right angular gyrus functional connectivity with FPCN (*p* = 0.046) (see Supplementary Table S2 strongest clusters peaks). However, these effects did not remain significant when differences in CFS signal between patients and Controls were controlled for (see Supplementary Methods: Control analyses).

The equivalent contrasts between rIFG lesion patients and Controls in the top two affected RNS revealed decreased functional connectivity between contralesional left supramaginal gyrus the FPCN in patients, relative to Controls (Fig. 4D, *p* = 0.025). We also observed increased coupling between the ipsilesional right angular gyrus and FPCN (*p* = 0.040) in rIFG patients compared to controls. However, this latter effect did not remain significant when differences between CFS signal between patients and Controls were controlled for (see Supplementary Methods: Control analyses). No regional clusters reached significance between rIFG patients and Controls for the other RSNs tested (see Supplementary Table S2 for strongest clusters peaks).

### Corroboration of functional connectivity using the Neurosynth database

To independently corroborate our functional connectivity results and to understand the wider connectivity of the lesion site in relation to the published literature, we seeded the SFG (− 18, 10, 50) and rIFG (32, 30, 6) lesion sites in the Neurosynth database (see Supplementary Methods: Neurosynth analysis and Supplementary Figure S3). Results suggest the SFG lesion site, as well as being connected to its contralateral homologue, functionally connects to only two clusters in the brain, located in extrastriate lateral occipital cortex (Figure S3A and B, LOC; cluster = 62, peak *z* = 0.227, MNI: − 32 − 82 36; cluster = 49, peak *z* = 0.223, MNI: − 6 − 68 54); one of which lies 10 mm medially and dorsally to the cluster found here to differentially connect with the aDMN. The Neurosynth database shows that the SFG and this LOC region tend to co-activate in motor imagery (*z* = 3.79) and simulation (*z* = 4.95) studies.

By contrast, the rIFG, in addition to its contralateral counterpart, is functionally connected to the dorsomedial PFC (Figure S3C cluster = 1296, peak *z* = 0.328, MNI: 4 14 50), bilateral supramarginal gyrus (Figure S3D cluster = 688, peak *z* = 0.282, MNI: 62 − 32 36; cluster = 185, peak *z* = 0.231, MNI: − 60 − 36 28) and bilateral superior frontal gyrus (Figure S3E cluster = 440, peak *z* = 0.275, MNI: 36 46 32; cluster = 127, peak *z* = 0.23, MNI: − 36 44 36). Co-activation association analysis suggests that these regions tend to co-activate in pitch perception (*z* = 4.57), language (*z* = 3.81) and pain (*z* = 3.55) studies.

### Network parameters in healthy controls

To directly compare the relative connectivity of the SFG and rIFG, we quantified the WM and resting state fMRI connectivity in healthy Control participants using common summary measures of node connectivity (Alstott et al. 2009; Hwang et al. 2013), see Supplementary Methods: Network definition and parameter measures in healthy Controls. For WM connectivity, we calculated the total number of cortical and subcortical GM target ROIs reached by probabilistic tractography tracts seeded from the two lesion overlap sites (degree of connectivity). The results suggested that the IFG WM was more highly connected to the rest of the brain than the SFG WM site (Supplementary Figure S4B, degree *t*_16_ = − 2.75, *p* = 0.014). By contrast, in resting state data a partial correlation analysis of BOLD timeseries extracted from cortical and subcortical GM anatomical targets identified the number of significant paired regions (degree of connectivity) and the sum of the absolute partial correlation coefficient (strength of connectivity) for the SFG and rIFG seeded regions (Supplementary Figure S4C). Contrary to WM network metrics, both degree and strength metrics suggested that the SFG had greater network connectedness than the IFG (Degree *t*_17_ = 4.31, *p* < 0.001; Strength *t*_17_ = 4.00, *p* < 0.001, Supplementary Figure S4D and S4E, respectively).

## Discussion

This study looked for converging evidence of the effects of circumscribed damage on functional and structural networks. For practical reasons, in this proof-of-principle study, we focused on damage to two regions of the frontal lobes, the superior and inferior frontal gyrus, both implicated in cognitive control and behavioral inhibition (Aron et al. 2014; Geddes et al. 2014; Modirrousta and Fellows 2008; Rae et al. 2015; Tsuchida and Fellows 2009; Yeung et al. 2021) and overlapping with regions included in the simulated lesions by Alstott and colleagues (2009). GM differences were identified between patients and Controls as a first step, and we used complementary, orthogonal network analysis in healthy participants to define the normal networks. We then compared structural and functional connectivity between patients and Controls to identify network-wide differences in WM or GM identified as networked with the lesion site. We also tested predictions of the effects of lesions on brain organization derived from anatomically informed models of large-scale functional and structural connectivity (Alstott et al. 2009), aiming to resolve discrepancies in the existing lesion literature (Eldaief et al. 2017; Nomura et al. 2010). The results suggest that chronic focal damage may have had effects on distant parts of the brain in both frontal lobe groups, with the extent differing substantially depending on how the network was probed.

### Extent of lesion impact on brain networks depend on modality but not location

Our primary analysis examined the extent to which lesions affected distant GM, WM and RSNs. Alstott et al. (2009) made simulated lesions in regions overlapping with our SFG and IFG lesions in a computational model and examined changes in resting state and diffusion-derived networks. This yielded the prediction that functional connections would be more affected by lesions than structural connections. Our experimental results, however, do not support this hypothesis. Instead, we found relative preservation of both functional and structural network metrics in the lesion groups (discussed below). By contrast, GM appears to show the greatest difference between lesion groups and Control subjects: we found evidence of widespread differentiable effects in GM volume far beyond both local lesion sites, relative to healthy Controls. SFG damage was associated with large disruptions in GM volume in an extended superior-fronto-cortico-thalamic set of regions and rIFG damage was associated with reduced GM in a more inferior fronto-cortico-thalamic set of regions. One interpretation of these effects is that GM, which likely reflect transneuronal degeneration, is particularly sensitive to the impact of lesions and is likely causally altered by the chronic experience of focal damage. However, an alternative interpretation is that the connectivity of a region may predict its vulnerability to a lesion and that these GM differences and other small network reorganization were, to some extent, present before the lesion. In which case these differences may predict vulnerability sites for lesions and could therefore have potential valuable clinical implications. This avenue could only be investigated through large-scale longitudinal studies.

Also to note, our two lesion sites varied in their proximity to the cortical midline. According to Alstott’s model, midline lesions should have more widespread effects than lesions to lateral regions of cortex as midline areas are more likely to be cortical hubs (Alstott et al. 2009). In the present study, however, we find little evidence that medial lesions have greater impact than lateral lesions. While the SFG is a more widely interconnected node when measured with functional connectivity in healthy controls, the rIFG is more connected to other brain regions through WM tracts. We also see that lesions to either node affect structural network metrics (i.e., GM volume and fractional anisotropy) and functional metrics (dual regression) to a comparable degree. The only exception is that despite the SFG and rIFG lesions overlapping to a comparable extent with the DMN, only SFG lesions affected connectivity with this network. Consistent with this finding, midline mPFC lesions do not alter intrinsic functional connectivity among undamaged DMN nodes (Eldaief et al. 2017). Instead, like the SFG RSN effects reported here, network-specific changes manifest as weaker correlations between whole brain RSNs. Collectively, our work suggests that in the case of the SFG and rIFG as midline and lateral nodes, respectively, there is little difference between the global lesion impact. Future work in larger samples could combine the graph theory analysis frameworks with the current study methods to identify whether lesion sites belong to hubs.

### A nonlinear relationship between structural and functional networks

Overall, this comparative neuroimaging approach in the same participants provided little evidence that distal GM volume loss is underpinned by degeneration of intervening WM tracts. While transneuronal degeneration can be caused by a loss of signal input (Heimer and Kalil 1978) and relationships between GM and WM degeneration have been observed in people with Alzheimer’s disease (Jang et al. 2017), our study suggests this is not always the case. Indeed, GM atrophy, independent from WM damage has also been noted in multiple sclerosis patients (Zhang et al. 2021) and has been accounted for by the impact of other pathological processes, such as the inflammatory response. Similar processes may also contribute to GM atrophy in stroke patients (Beuker et al. 2022; Dharmajaya and Sari 2021), with such secondary tissue damage known to impede recovery (Iadecola and Anrather 2011; Moskowitz et al. 2010). Ultimately, the GM degeneration in the present study appeared at sites known to the connected structurally and functionally (in non-brain damaged controls) to the lesion sites although given the limitation of the imaging methods we are only able to examine the macroscopic scale (Silasi and Murphy 2014). Given our cross-sectional design, we are prevented from drawing inferences about reorganization across time or their relationship to clinical outcomes. These latter measures are only possible through longitudinal neuroimaging and behavioral studies. In the present study, only the corpus callosum and internal capsule (predominantly) the cerebral peduncle and corticospinal pathways) showed reduced structural integrity in SFG patients relative to Controls, while rIFG lesions only affected WM metrics in the internal capsule (with tractography suggesting the affected fibers project to thalamic frontal tracts and EmC) as well as projecting through ILF and IFOF, UF tracts) (Schmahmann and Pandya 2006). This pattern of effects may indicate that despite lesion heterogeneity, WM involved in motor control is most vulnerable at the chronic lesion stage. The different measures may therefore show effects at different time scales (Berthier et al. ﻿^2011^).

We also found little evidence that GM degeneration affects functional connectivity. Although some recent work has linked RSNs and GM metrics in healthy young people (Hunt et al. 2016) and people with neurological conditions, such as Parkinson’s Disease (Lucas-Jimenez et al. 2016), our results suggest that chronic focal lesion effects on distant GM regions are only accompanied by small-scale changes in functional connectivity within well-established RSNs. This finding is consistent with an in silico lesion model that showed human functional brain networks are relatively resilient to extensive damage (Joyce et al. 2013). We identified both reduced and increased connectivity in patient groups relative to Controls. For example, in SFG patients we found that lateral occipital cortex connectivity with the aDMN was significantly reduced compared to Controls, while increased connectivity was found between aDMN and rostral frontopolar cortex. A similar pattern was seen in the rIFG patients, with reduced functional coupling between FPCN and contralesional supramarginal gyrus, but increased coupling with the ipsilesional angular gyrus. This overall pattern of network preservation begs the question: How can functional networks be so globally robust to lesions which affect network nodes? This question is further complicated by evidence that nodes have unequal status (van den Heuvel and Sporns 2013). One possibility is that the RSNs studied here (at least) emerge due to common (perhaps subcortical) drivers rather than cortico-cortical structural connections.

### Limitations and future directions

Analysis of multiple types of imaging comes with a number of challenges and limitations. For example, the degrees of freedom within analysis methodology could lead to false positives if enough analyses are run. To reduce this possibility, we constrained our analyses to the most widely used and validated platforms within the FSL toolbox. While an alternative analysis approach might have included more formal graph theory and connectome methods, we note that interpreting such rich data is often difficult, and it is particularly challenging to interpret likely holistic impact on patients themselves. While we do not compare directly across data types because of limited sample size, future studies with more power may also subject this type of data to exploratory analysis tools designed to automatically find patterns of effects consistent across imaging modalities (Groves et al. 2011). Furthermore, future studies should include task-related FC measures as well as resting state FC, as dynamic diaschisis has been described following injury with differences only apparent in certain contexts (Price et al. 2001). Our study also does not probe the potential relationship between functional/structural connectivity and behavior (Siegel et al. 2018). Finally, patient data itself provide additional challenges. For example, some lesions may pose problems for standard segmentation and registration software. To minimize this possibility, we meticulously checked segmentation and registration images and confirmed no outliers drove the group differences. Further, in the absence of longitudinal measures it is challenging to ascertain whether the findings we observed in the chronic stage are secondary to a pathological state (diaschisis) or arise as compensatory mechanisms during recovery (Carrera and Tononi 2014). Our study does not test the effect of lesion chronicity on network connectivity. Longitudinal data accompanied by behavioral measures will be needed to address these important questions (see Fornito et al. 2015 for discussion).

## Conclusions

In this proof-of-principle study, we examined the effects of focal damage to two regions of the frontal lobes on structural and functional networks. Extensive differences in GM volume were evident beyond the lesion site, relative to Controls, but WM paths and functional networks were largely conserved. Some limited functional connectivity differences were found in resting state networks thought to underpin higher-order cognitive processes, but WM differences were only detected in cortico-motor pathways. The findings shed light on the potential neural substrates of widely studied RSNs, showing unexpected discordance between different structural and functional measures and also can be used to refine existing computational models of such brain networks.

### Electronic supplementary material

Below is the link to the electronic supplementary material.

Supplementary file1 (DOCX 2544 KB)

## Data Availability

The datasets generated during and/or analyzed during the current study are not publicly available due as participants did not consent to public release but are available from the corresponding author on reasonable request.

## Appendix

Harvard Oxford Atlas (See Table 3).

**Table 3:** 56 cortical and sub-cortical regions used to construct structural networks as defined by the Harvard–Oxford atlas

Label	Anatomical region	Label	Anatomical region
1	Frontal pole (FPole)	29	Cingulate gyrus AD (ACG)
2	Insular ctx (IC)	30	Cingulate gyrus PD (PCG)
3	Superior frontal gyrus (SFG)	31	Precuneous ctx (PCUN)
4	Middle frontal gyrus (MFG)	32	Cuneal cortex (CUN)
5	Inferior frontal gyrus, pars triangularis (IFGtriang)	33	Frontal orbital ctx (Forb)
6	Inferior frontal gyrus, pars opercularis (IFGoper)	34	Parahippocampal gyrus (PHIPant)
7	Precentral gyrus (PreC)	35	Parahippocampal gyrus (PHIPpost)
8	Temporal pole (TPole)	36	Lingual gyrus (LIN)
9	Superior temporal gyrus AD (STGant)	37	Temporal fusiform ctx (TFUSant)
10	Superior temporal gyrus PD (STGpost)	38	Temporal fusiform ctx (TFUSpost)
11	Middle temporal gyrus AD (MTGant)	39	Temporal occipital fusiform ctx (TOFus)
12	Middle temporal gyrus PD (MTGpost)	40	Occipital fusiform gyrus (OFus)
13	Middle temporal gyrus, TOP (MTGto)	41	Frontal operculum ctx (FOper)
14	Inferior temporal gyrus AD (ITGant)	42	Central opercular ctx (COper)
15	Inferior temporal gyrus PD (ITGpost)	43	Parietal operculum ctx (POper)
16	Inferior temporal gyrus, TOP (ITGto)	44	Planum polare (PPolare)
17	Postcentral gyrus (PostC)	45	Heschls gyrus (HES)
18	Superior parietal lobule (SPL)	46	Planum temporale (PTemporale)
19	Supramarginal gyrus AD (SMGant)	47	Supracalcarine ctx (SupraCAL)
20	Supramarginal gyrus PD (SMGpost)	48	Occipital pole (Opole)
21	Angular gyrus (ANG)	49	Thalamus (THA)
22	Lateral occipital ctx SD (LOCsup)	50	Caudate (CAU)
23	Lateral occipital ctx ID (LOCinf)	51	Putamen (PUT)
24	Intracalcarine ctx (IntraCAL)	52	Pallidum (PAL)
25	Frontal medial ctx (FMC)	53	Brain stem (BS)
26	Juxtapositional ctx (SMA)	54	Hippocampus (HIP)
27	Subcallosal ctx (subcallosal)	55	Amygdala (AMYG)
28	Paracingulate gyrus (ParaCG)	56	Accumbens (NAC)

*AD* anterior division, *PD* posterior division, *SD* superior division, *ID* inferior division, *TOP* temporooccipital part, *ctx* cortex
