# Supplementary material for: Characterization of structural and functional network organization after focal prefrontal lesions in humans in proof of principle study
Source: Brain Struct Funct. 2022 Oct 7;227(9):3027–41. doi: 10.1007/s00429-022-02570-2 (PMC9653359; doi:10.1007/s00429-022-02570-2)
Supplement: Supplementary file 1 — Supplementary file1 (DOCX 2544 KB) [file 429_2022_2570_MOESM1_ESM.docx]

# Supplementary Methods

To quantify the VBM effects and inform our interpretation, we examined connectivity of the two lesion sites in the healthy Control group, as well as a non-lesioned control site. We therefore conducted two additional connectivity analyses in the healthy control sample: probabilistic tractography and seeded resting state.

## Probabilistic tractography: From lesion site to VBM effects

In healthy Control subjects, we defined the structural connectivity between the lesioned brain areas and the VBM lesion effects. The lesion overlap sites (SFG MNI -15, 11, 50 and rIFG MNI 28,31,11, see Results: [Patient Characteristics](#_Participant_characteristics) for more detail) were used as WM ROI seeds (216mm^3^), registered to individual subject space, in a probabilistic tractography analysis in healthy Controls. Voxel-wise estimates of the fiber orientation distribution were calculated using Bedpostx, limited to estimating two fiber orientations at each voxel, because of the b value and number of gradient orientations in the diffusion data (Behrens, Berg et al. 2007 http://fsl.fmrib.ox.ac.uk/fsl/fslwiki/FDT). Probabilistic tractography was run for each subject, using a model accounting for multiple fiber orientations in each voxel. Five thousand sample streamlines were seeded from each voxel within each individual’s seed mask. The tractography algorithm parameters used were a maximum of 2000 steps; step size of 0.5 mm and a curvature threshold of 0.2. Each streamline followed local orientations sampled from the posterior distribution given by BedpostX, as described previously. As a control site, and to reduce the possibility of biasing the results if one lesion site had a broader connectivity pattern than another, we also ran probabilistic tractography from the lateral occipital fusiform gyrus (LOFG, -14,-74,-12). The significant VBM effects acted as classification targets with probtrackx quantifying the connectivity values between the WM ROI seed mask (SFG, rIFG and LOFG) and the GM target mask (SFG lesion VBM effects and rIFG lesion VBM effects). The values in the resulting image file represent the number of samples seeded from that voxel reaching the relevant target mask voxels. We summed the connectivity values across GM seed voxels for each subject and normalised by the product of the size of the seed mask. We contrasted the degree of connectivity between each WM seed (SFG and IFG) and their respective lesion VBM effects against the same metrics derived from the control seed (ie [1] SFG WM seed –> SFG VBM vs LOFG WM seed –> SFG VBM and [2] IFG WM seed –> IFG VBM vs LOFG WM seed –> IFG VBM). Between subject differences in connectivity were quantified using paired-samples t tests.

Visitation maps referred to as tractograms were constructed for each individual from the raw output connectivity distribution (i.e., unconstrained by VBM effects). These connectivity distribution values were log transformed, normalized by dividing by the maximum tracts identified for each subject, thresholded at 0.8 and binarized (Mars, Foxley et al. 2015). We sought only the top 20% of tracts emanating from the seed mask. Finally, the tracts were summed across subjects, registered to MNI space and are illustrated thresholded at more than 50% of subjects. These tracts were used to constrain the lesion TBSS analyses described in the main manuscript, statistically acting as a small volume of interest.

Results are described in Results: [Connectivity of the SFG and IFG in healthy Controls](#_Connectivity_of_the)  and shown in [figure S1](#_Figure_S2.).

## Seeded resting state: From lesion site to VBM effects

In healthy Control subjects, we defined the networks based on the functional connectivity of the two anatomical regions identified in the patient sample. A 216 mm^3^ mask was drawn over the GM voxel closest (as judged by eye) to the centre of the lesion overlap. In the SFG group, the mask was drawn at MNI coordinates -18,10,50. According to the Harvard-Oxford cortical atlas this area of GM lies within the superior frontal gyrus, although closely flanked by middle frontal gyrus, paracingulate gyrus and juxtapositional cortex. Neubert and colleagues refer to this region as pre Suplementary Motor Area (Neubert, Mars et al. 2015). Similarly, in the IFG group, we placed a 216mm^3^ mask slightly lateral and ventral to the rIFG WM coordinates in the nearest GM voxel at MNI coordinates 32,30,6. According to the Harvard-Oxford cortical atlas this area of GM is at the intersection of the inferior frontal gyrus, orbitofrontal cortex, insular cortex and frontopolar cortex. Neubert and colleagues refer to this region as area 45a (Neubert, Mars et al. 2014). As a control site, we placed a 216mm^3^ mask in the lateral occipital fusiform gyrus (LOFG, MNI: -18,-74,-12). Cortex here was not damaged in either patient group. From these masks, the BOLD time series from all the healthy Control subjects was extracted and used as an explanatory variable in a primary GLM analysis (FEAT). A nuisance regressor of the signal time series of the whole brain was also included in each primary-level analysis. Mixed effects analyses (FLAME 1 and 2) were applied to the whole brain group data in MNI space to generate statistical activation maps for each of the contrasts and to test for an effect of group. Group Z (Gaussianized t) statistic images were thresholded using clusters determined by *Z* = 2.3 and a corrected cluster extent significance threshold of *p* = 0.05. We then compared the spatial topography of the group-level seeded resting state networks with that of the VBM lesion effects (including lesioned voxels).

To quantify the degree of overlap we used the Harvard Oxford parcellation atlas. We indexed the seeded resting state (sRS) network by seeking those ROIs where group-wise significant sRS effects covered more than 25% of the ROI. Similarly, we indexed the VBM network by seeking those ROIs where significant VBM effects covered more than 25% of the ROI. We discounted any ROIs in which 50% of the voxels were damaged in any lesion patient. Overlap was then calculated as the proportion of ROIs in which the VBM effects overlapped with the sRS effects. This VBM x sRS overlap was also used, in part, to constrain the lesion dual regression analysis described in the main manuscript, statistically acting as a small volume of interest.

Results are described in Results: [Connectivity of the SFG and IFG in healthy Controls](#_Connectivity_of_the)  and shown in [figure 2](#Figure 2. Seeded resting state analysis in healthy Controls seeded from centre of gravity of the overlap shown of (A) seven SFG patients and (B) six rIFG patients. The SFG seed was moved laterally into the closest GM shown overlapped (red) with the SFG VB), [figure S2](#_Figure_S3.) and [table S1](#_Table_S1._1).

## Neurosynth analyses

The Brain Genomics Superstruct Project within the neurosynth (http://neurosynth.org/) interface was used to create a map which represents resting-state functional connectivity analysis performed on 1,000 human subjects. We placed two seeds in GM voxels within the SFG (-18,10,50) and rIFG (32,30,6) lesion site. Functional connectivity maps were thresholded at z > 0.2 and cluster extent above 100 voxels. Neurosynth meta-analytic coactivation maps were also generated from 6mm diameter spheres centred on the same voxel locations. Values represent z-scores quantifying the strength of association between the presence or absence of activation in each voxel in relation to the presence or absence of activation in the seed voxel. We report the highest-ranking z-scores for non-anatomy co-activation association terms. These represent the z-score value obtained at the current voxel in the "association test" meta-analysis map for the corresponding term.

Results are reported in Results: [Corroboration of functional connectivity using the Neurosynth database](#_Corroboration_of_functional) and [figure S3](#_Corroboration_of_functional).

## Network definition and parameter measures in healthy controls

Using DMRI and resting state data from healthy Controls, we defined the normal network of each lesion network and estimated network connectedness. Findings are reported in the main manuscript (*Results:* [*Defining the lesion site*](#_Defining_the_lesion) *and* [*Network parameters in healthy Controls*](#_Network_parameters_in)) and shown in Supplementary [figure S4](#_Figure_S1.).

First, in healthy Controls, we defined the structural WM connectivity between the regions damaged in the lesion sample. The lesion overlap clusters identified above were registered to individual subject space and used as seeds (216mm^3^) in a probabilistic tractography analysis in healthy Controls using the same parameters described above.

Instead of the VBM effects acting as classification targets, in this analysis connectivity was estimated from each voxel within the two WM ROI seeds to every voxel in the brain. Each resulting connectivity weight was then categorized using the Harvard-Oxford cortical and subcortical atlases offering a partitioning of each hemisphere into 56 anatomically distinct regions– 48 cortical and 8 subcortical. For each subject, connectivity weights between each WM ROI seed voxel to any voxel in each of the 56 anatomical target ROIs were summed across seed voxels and the peak connectivity value within each target ROI was extracted and averaged across subjects. This analysis provided data-driven confirmation of our lesion grouping (see *Results:* [*Defining the lesion site*](#_Defining_the_lesion)*; Supplementary* [*Figure S1*](#_Figure_S2.)). In all subjects, we also calculated the total number of target ROIs reached by any tract, thresholded above 25% of the maximum tracts reached by any subject to any area for each seeded location (nodal degree), and compared the two WM ROI-seeded analyses with paired samples t-tests. As the 25% threshold is arbitrary, we also report these statistics for the flanking thresholds of 10%, 20%, 30% and 40%.

Second, we estimated network parameters from resting state data through a partial correlation analysis. Parcellations from Harvard Oxford cortical and subcortical atlas were used as anatomical ROIs. Anatomical ROI masks were registered to each healthy control’s MRI scan and fMRI scan space in a step-wise manner, and the BOLD time series were extracted from each mask in each subject. We partialled out the confounding influence of the whole brain GM, WM, and cerebrospinal fluid (CSF) BOLD time courses by using the FSL general linear model (GLM) tool. We focused on coupling between the Harvard-Oxford SFG and IFG parcellations and each of the other 55 anatomical regions. The time series for pairs of regions were entered into four partial correlation analyses that each controlled for the correlation with the BOLD time series in all 55 other ROIs. Network parameters, degree, defined as the number of links connected to the node, and strength, defined as the sum of weights of outward links connected to the node, were calculated on a subject-wise basis as the sum of the significantly paired anatomical regions and the sum of the absolute partial correlation coefficient respectively. Paired t tests examined the between group difference in degree and strength. The resulting partial correlation values were then Fisher transformed and entered into a correlation and compared against zero in multiple Bonferroni-corrected t tests.

## Control analyses

Whole brain parameters of intracranial volume and frame displacement (an index of movement) were measured as the total number of voxels in each subject’s structural scan and the sum of frame-wise movement estimated from the resting state data during FSL pre-processing. Lesion groups were compared against controls using independent samples t-tests. Lesion groups were also compared on the number of years since lesion onset with independent samples t-tests. Finally, we examined whether differences in global signal explained some of our key dual regression effects. First, we extracted the CFS timeseries from all subjects standardised filtered denoised EPI data and compared the median value of each subjects’ timeseries across groups in two between-subjects t-tests.

# Supplementary Results

## Lesion overlaps

The lesion overlap image files for the SFG and rIFG groups are available. They are titled SFGLesionOverlap.nii.gz and rIFGLesionOverlap.nii.gz respectively.

*Extended differences in structural morphometry beyond the lesion sites*

Contrast maps of the VBM analyses that show greater grey matter in healthy Controls than SFG lesion patients can be found in the supplementary image file titled VBM_Control>SFG_Corrp_tstat.nii.gz. Greater grey matter in healthy Controls relative to rIFG lesion patients can be found in found in VBM_Control>rIFG_Corrp_tstat.nii.gz.

## Probabilistic Tractography structural connectivity from the SFG and IFG in healthy controls


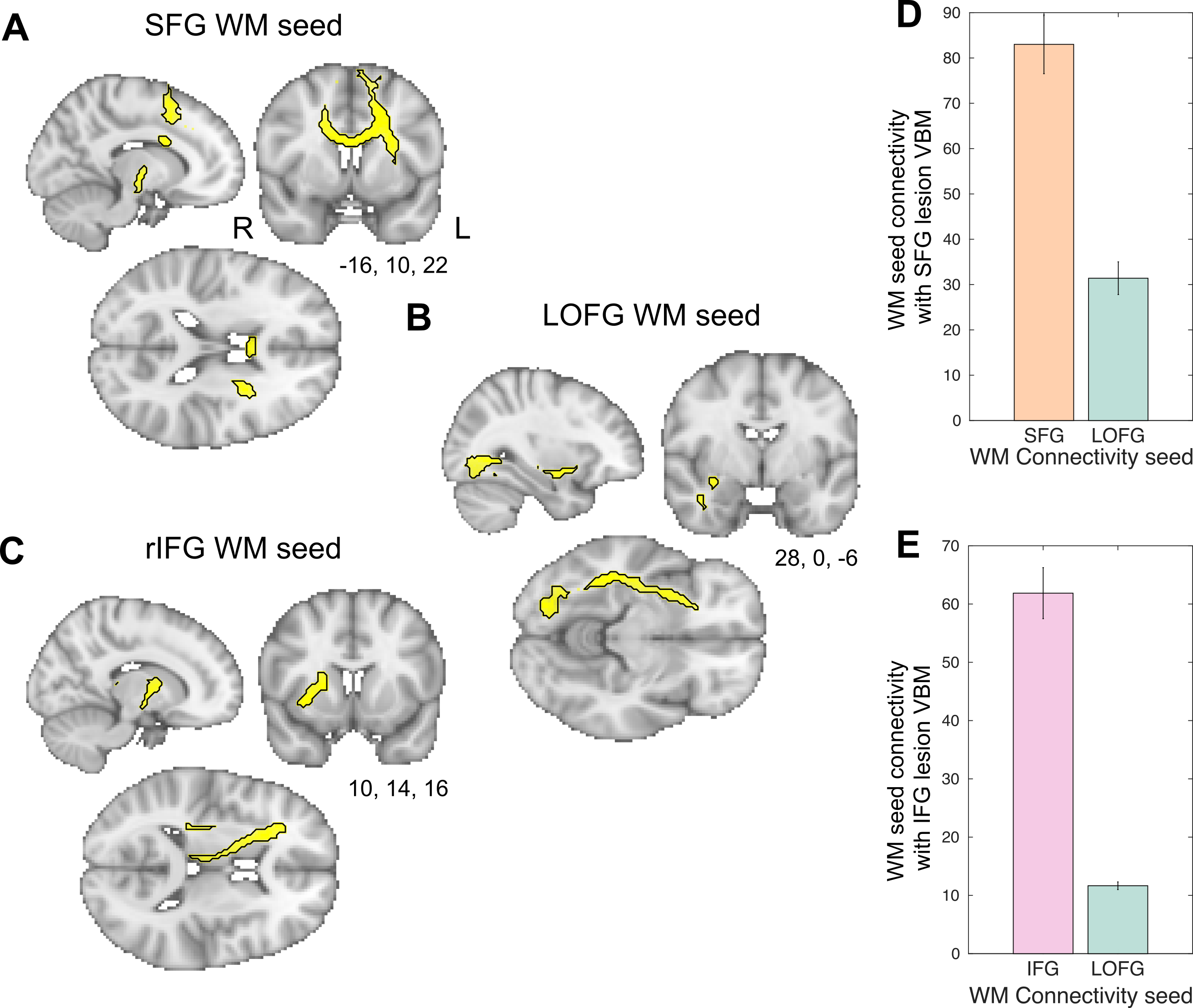


### Figure S1. Probabilistic tractography in healthy Controls seeded from a 3mm^3^ ROI at the coordinates of the SFG or rIFG lesion most commonly affected in the patient sample (A and B respectively). Tractography seeded in the SFG courses through corpus callosum and corticospinal tracts, while the rIFG seed passes through the Extreme Capsule (C) Probabilistic tractography in healthy controls seeded from a 3mm^3^ ROI in the control LOFG region. The control LOFG seed is part of the inferior longitudinal fasciculus and uncinate fasciculus. (D-E) Sum and standard error of probabilistic tractography connectivity weights in healthy controls between WM ROI seed and target VBM lesion effects comparing each WM lesion seed to the WM control seed.

## Seeded resting state functional connectivity from the SFG, IFG and LOFG in healthy controls


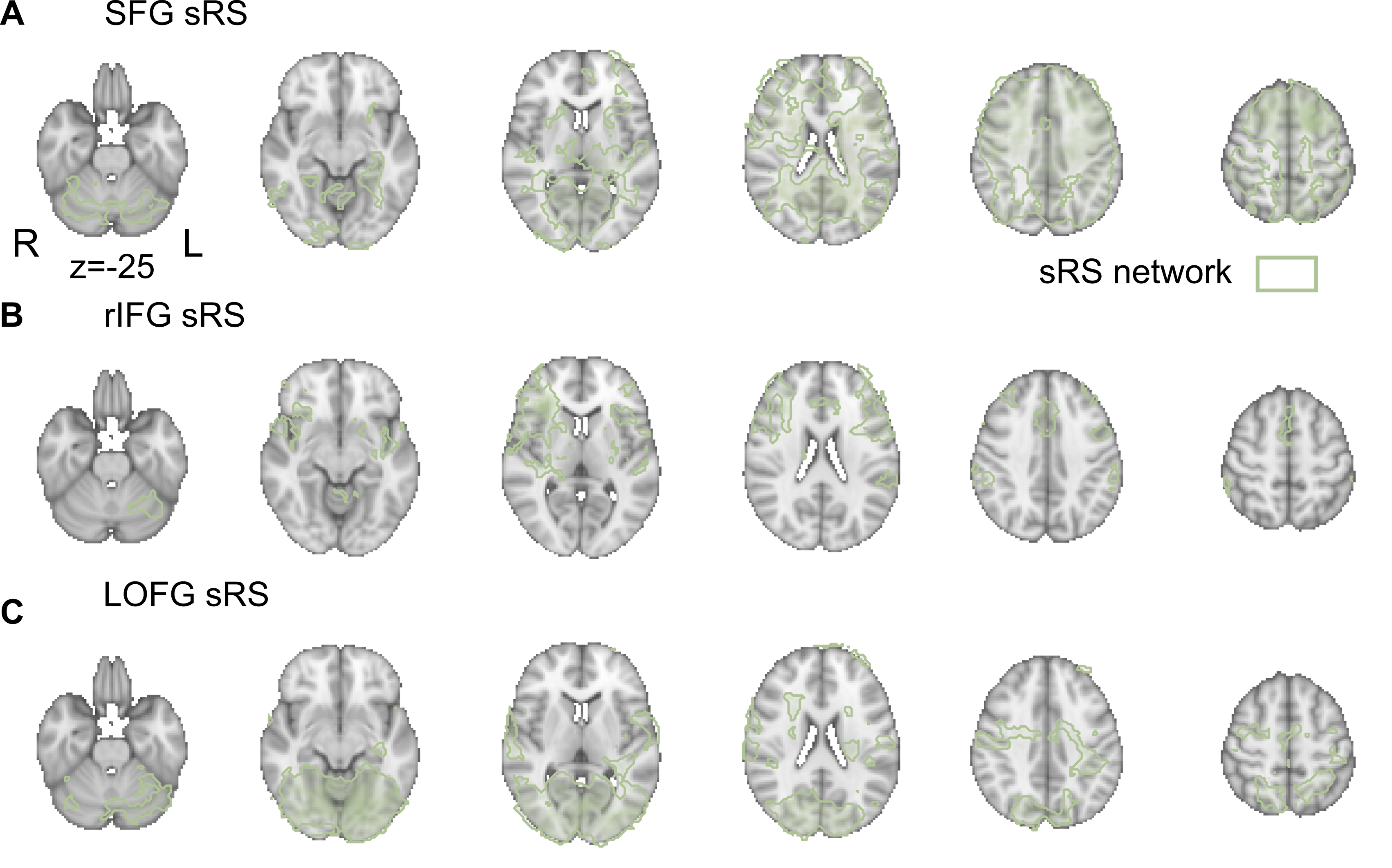


### Figure S2. Seeded resting state analysis in healthy Controls seeded from centre of gravity of the overlap shown of (A) seven SFG patients and (B) six rIFG patients. SFG seed was moved laterally into the closest GM voxel. rIFG seed was moved laterally and ventrally into the closest GM voxels. C shows the same resting state analysis in Controls seeded from the lateral occipital fusiform gyrus (LOFG) as a control region. Brain slices increase in intervals of 14 mm from the most ventral slice of z = -25.

## Quantification of seeded resting state functional connectivity from the SFG, IFG and LOFG in healthy controls with SFG and rIFG VBM lesion effects.

### Table S1. Harvard Oxford ROIs identified in which VBM lesion effects overlap with the seeded resting state networks. This excludes any ROI with more than 50% lesion damage. Abbreviations in Appendix.

| ***SFG VBM*** | | | | ***IFG VBM*** | | | |
| --- | --- | --- | --- | --- | --- | --- | --- |
| ***SFG sRS*** | | ***LOFG sRS*** | | ***IFG sRS*** | | ***LOFG sRS*** | |
| ***Region*** | ***num voxel overlap*** | ***Region*** | ***num voxel overlap*** | ***Region*** | ***num voxel overlap*** | ***Region*** | ***num voxel overlap*** |
| rMFG | 1700 | rLOCinf | 886 | lParaCG | 907 | rSTGpost | 561 |
| rPreC | 1995 | rLIN | 455 | lIC | 438 | rPostC | 917 |
| rParaCG | 742 | rTOFus | 316 | lSMGant | 421 | rSMGant | 474 |
| rPCG | 301 | lLOCsup | 1345 | lPOper | 222 | rLOCsup | 1557 |
| rLIN | 455 | lCOper | 412 | lPPolare | 292 | rLOCinf | 807 |
| rTHA | 1031 | lPOper | 169 | lPTemporale | 189 | rPTemporale | 291 |
| lFPole | 2996 | lPTemporale | 232 | lPUT | 749 | rOpole | 899 |
| lIC | 942 |  |  | lNAC | 56 | lLOCsup | 1358 |
| lIFGtriang | 419 |  |  |  |  | lLOCinf | 652 |
| lIFGoper | 345 |  |  |  |  | lCOper | 512 |
| lPreC | 1534 |  |  |  |  | lPOper | 222 |
| lPostC | 1254 |  |  |  |  | lPTemporale | 189 |
| lSMGant | 716 |  |  |  |  | lOpole | 755 |
| lSMGpost | 541 |  |  |  |  |  |  |
| lLOCsup | 1345 |  |  |  |  |  |  |
| lPCG | 565 |  |  |  |  |  |  |
| lFOper | 244 |  |  |  |  |  |  |
| lCOper | 412 |  |  |  |  |  |  |
| lHES | 235 |  |  |  |  |  |  |
| lTHA | 1138 |  |  |  |  |  |  |
| lCAU | 427 |  |  |  |  |  |  |
| lPUT | 799 |  |  |  |  |  |  |

## Corroboration of functional connectivity using the Neurosynth database

*
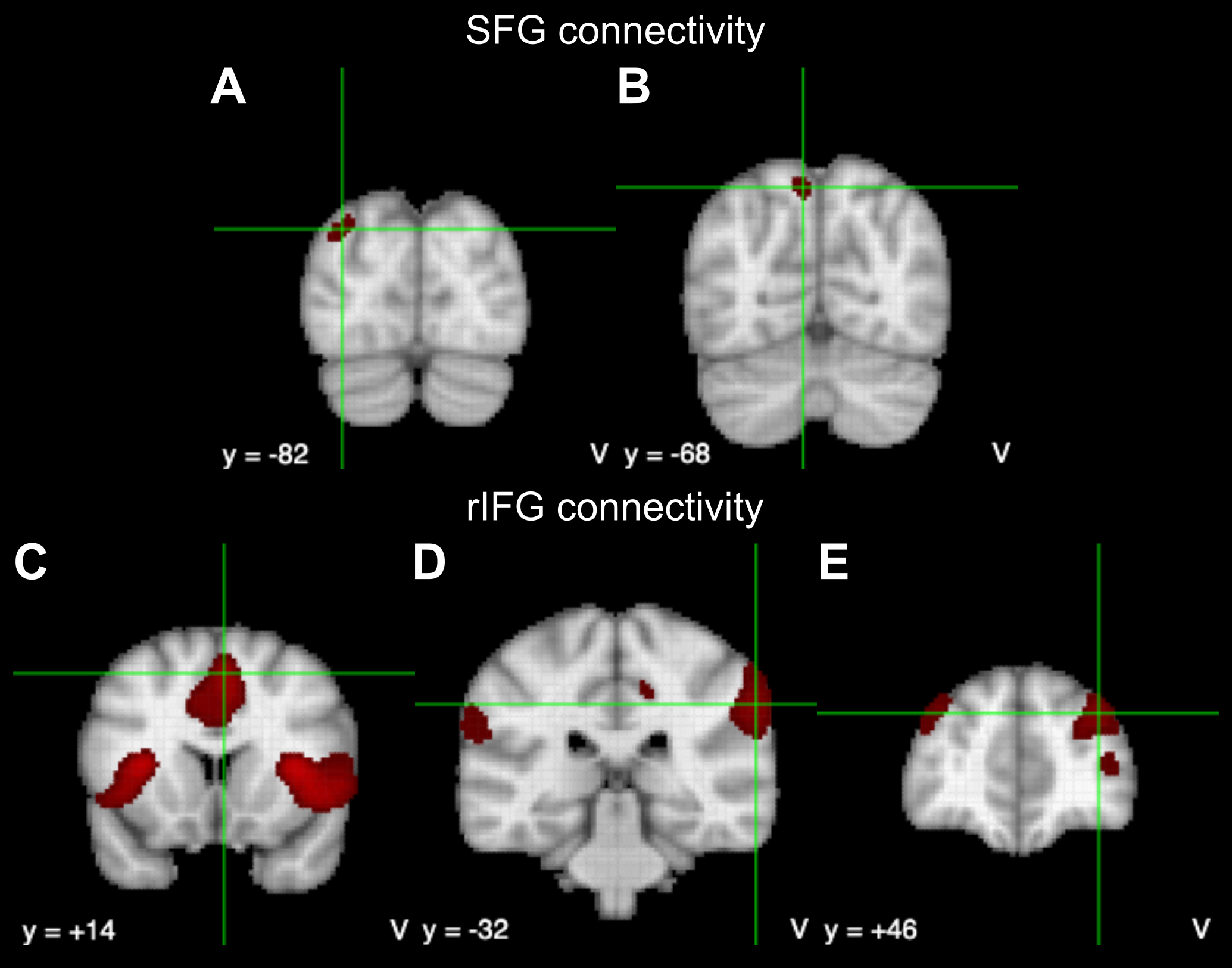
*

### Figure S3. Resting-state functional connectivity for a seed region in SFG (MNI -18, 10, 50) and rIFG (MNI 32, 30, 6) in the Neurosynth database. The SFG functionally connects to two clusters in extrastriate lateral occipital cortex (A and B). The rIFG functionally connects to the dorsomedial PFC (C), bilateral supramarginal gyrus (D) and bilateral superior frontal gyrus (E). Images thresholded between -0.2 and 0.2. Cross hairs placed on peak coordinates. Where bilateral regions were identified the coordinate is placed in the larger of the two clusters.

## Limited differences in resting state networks after frontal lobe lesions

### Table S2. Dual regression effects. Reporting p values of the strongest clusters, cluster size and corresponding peak MNI coordinates.

|  |  | **Control>lesion** | | | **Lesion>control** | | |
| --- | --- | --- | --- | --- | --- | --- | --- |
| **Lesion** | **RSN** | **P value** | **Num voxels** | **MNI coord** | **P value** | **Num voxels** | **MNI coord** |
| **SFG** | **aDMN** | 0.006* | 36 | -42, -86, 20  LOC | 0.013* | 47 | 30, 70, 12  FPC |
|  | **SMN** | 0.186 | 17 | - | 0.304 | 14 | - |
|  | **VAN** | 0.327 | 15 | - | 0.026*^+^ | 39 | -42 -18 4  IC |
|  | **DAN** | 0.074 | 22 | - | 0.364 | 12 | - |
|  | **VisN** | 0.491 | 11 | - | 0.223 | 22 | - |
|  | **FPCN** | 0.178 | 19 | - | 0.046*^+^ | 30 | 54 -46 52  AG |
| **rIFG** | **FPCN** | 0.025* | 25 | -54,-38, 24  SMG | 0.04*^+^ | 33 | 50, -46, 36  AG |
|  | **DMN** | 0.806 | 5 | - | 0.167 | 20 | - |
|  | **VAN** | 0.831 | 5 | - | 0.329 | 17 | - |
|  | **VisN** | 0.078 | 17 | - | 0.473 | 11 | - |
|  | **DAN** | 0.375 | 12 | - | 0.795 | 6 | - |
|  | **SMN** | 0.562 | 7 | - | 0.054 | 23 | - |

* p < 0.05,

^+^ not significant once differences in CFS between patients and controls are accounted for.

Abbreviations: lateral occipital cortex (LOC), Frontopolar cortex (FPC), Insula cortex (IC), Angular gyrus (AG), Supramarginal gyrus (SMG).

## Network definition and parameters


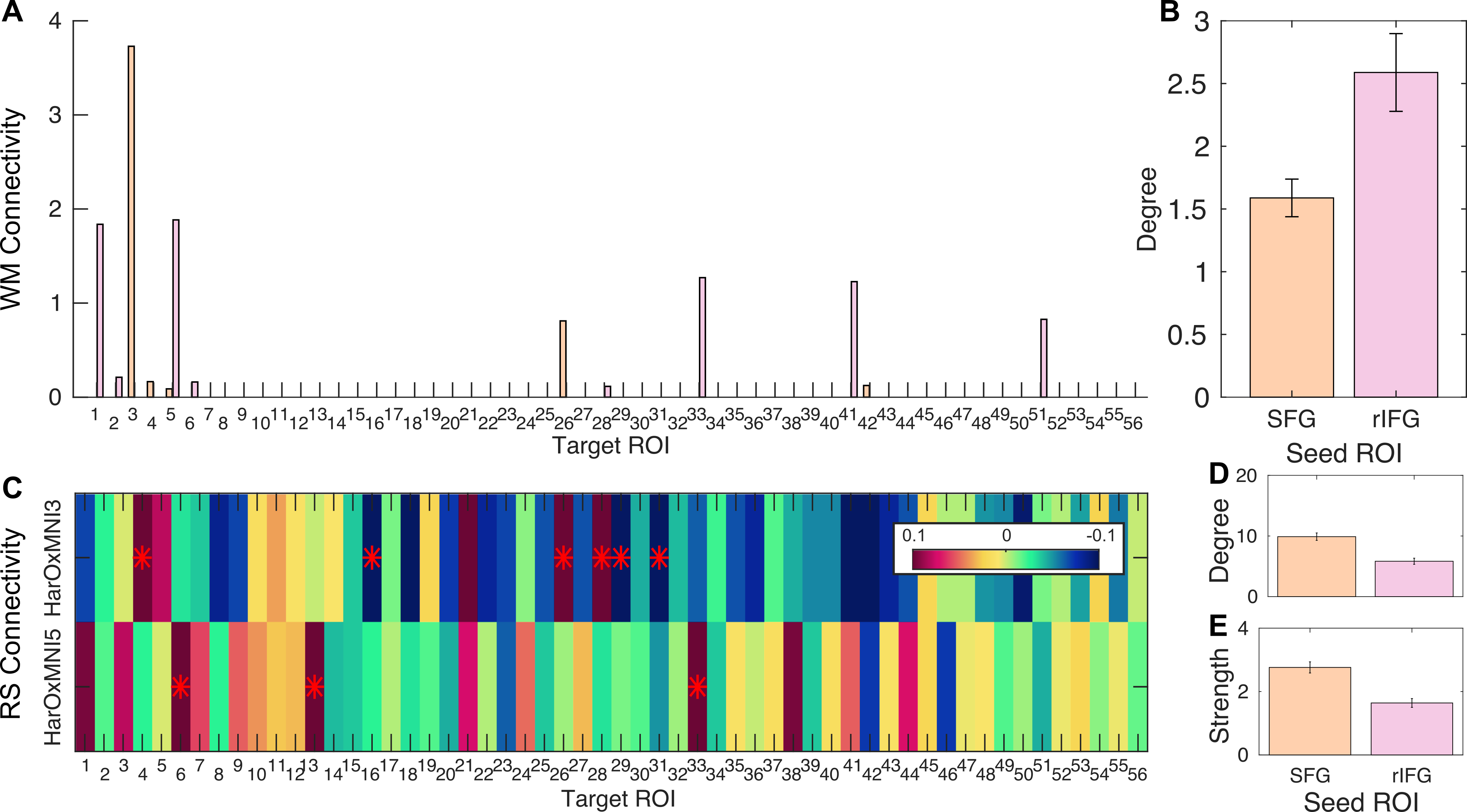


### Figure S4. Defining the lesion network in healthy controls. A. Mean of the maximum WM connectivity weight for each participant for each of the 56 Harvard Oxford parcellation target ROIs reached by WM ROI seeded tractography at the two lesion sites. B. Total number of target ROIs, regardless of weight, reached by WM ROI seeded tractography at the two lesion sites. For illustration purposes, connections have been thresholded above 25% of the maximum tracts reached by any subject to any area for each seeded location. We also compared the degree of WM connectivity between SFG and rIFG seed sites at a range of thresholds. Removing 10% and 20% of the maximum number of tracts (which likely reflect spurious connections) showed that rIFG had high connectivity to the rest of the brain than the SFG (10% threshold t_16_ = -8.35, p < 0.001, 20% threshold t_16_ = -3.78, p = 0.002), however, at higher thresholds this pattern became less significant (30% threshold t_16_ = -1.95, p = 0.069, 40% threshold t_16_ = -1.33, p = 0.203). C. Mean Fisher-transformed correlation coefficient representing the strength of BOLD coupling between each Harvard-Oxford parcellation ROI and the SFG and rIFG parcellation. D. Degree of resting state connectivity representing the mean total number of target ROIs in which BOLD activity is significantly coupled with the SFG and rIFG anatomical ROIs. Contrary to WM network metrics, connectivity degree as measured by resting state showed SFG had greater network connectedness than the IFG (Degree t_17_ =4.31, p < 0.001). E. Strength of connectivity representing the mean total number of target ROIs significantly paired with the SFG and rIFG anatomical ROIs. Similarly, connectivity strength measured by resting state suggested that the SFG was more connected that the rIFG (Strength t_17_ = 4.00, p < 0.001).

## Control analyses

We performed a number of control analyses to rule out involvement of confound variables (se*e Supplementary Methods:* [*Control analyses*](#_Control_analyses)). Lesion groups showed no differences in whole brain parameters of intracranial volume (ts =< -1.97, ps=>0.061) or frame displacement, an index of movement during the scan (ts =< 0.34, ps => 0.732) compared to Controls, arguing that these confounds are unlikely to explain the observed effects. Furthermore, differences in global signal, potentially reflecting alterations in cerebral vasculature also could not explain all our effects. When this signal was regressed out of the dual regression effects both of the DMN effects remained significant (Controls>SFG t_23_ = 2.57, p = 0.017 [Figure 4B](#Figure 4. Lesion effects on resting state networks (RSNs) functional connectivity. (A) Proportion of voxels that overlap between of each of the six Yeo RSNs and each lesion patients lesion mask for with the SFG (peach) and rIFG (pink) respectively. The SF), SFG>Controls t_23_ = -3.11, p = 0.005 [Figure 4C](#Figure 4. Lesion effects on resting state networks (RSNs) functional connectivity. (A) Proportion of voxels that overlap between of each of the six Yeo RSNs and each lesion patients lesion mask for with the SFG (peach) and rIFG (pink) respectively. The SF)). Similarly, the decreased functional connectivity between FPCN and left supramaginal gyrus cortex in rIFG patients compared to controls also remained significant after CFS signal was removed (Controls>rIFG t_22_ = 3.66, p = 0.001 [Figure 4D](#Figure 4. Lesion effects on resting state networks (RSNs) functional connectivity. (A) Proportion of voxels that overlap between of each of the six Yeo RSNs and each lesion patients lesion mask for with the SFG (peach) and rIFG (pink) respectively. The SF)). However, the other effects did not remain significant (SFG>Controls insula cortex-VAN t_23_, = -2.00, p = 0.063, SFG>Controls angular gyrus-FPCN t_23_, = -1.02, p = 0.320, rIFG>Controls angular gyrus-FPCN t_22_ = -1.93, p = 0.067).
